# Diversity and spoilage potential of microbial communities associated with grape sour rot in eastern coastal areas of China

**DOI:** 10.7717/peerj.9376

**Published:** 2020-06-16

**Authors:** Huanhuan Gao, Xiangtian Yin, Xilong Jiang, Hongmei Shi, Yang Yang, Chaoping Wang, Xiaoyan Dai, Yingchun Chen, Xinying Wu

**Affiliations:** 1 Shandong Academy of Grape, Jinan, China; 2 Shandong Academy of Agricultural Sciences, Institute of Plant Protection, Jinan, China

**Keywords:** Grape, High-throughput sequencing, Bacteria, Pathogenicity, Fungus

## Abstract

As a polymicrobial disease, sour rot decreases grape berry yield and wine quality. The diversity of microbial communities in sour rot-affected grapes depends on the cultivation site, but the microbes responsible for this disease in eastern coastal China, has not been reported. To identify the microbes that cause sour grape rot in this important grape-producing region, the diversity and abundance of bacteria and fungi were assessed by metagenomic analysis and cultivation-dependent techniques. A total of 15 bacteria and 10 fungi were isolated from sour rot-affected grapes. High-throughput sequencing of PCR-amplicons generated from diseased grapes revealed 1343 OTUs of bacteria and 1038 OTUs of fungi. *Proteobacteria* and *Firmicutes* were dominant phyla among the 19 bacterial phyla identified. *Ascomycota* was the dominant fungal phylum and the fungi *Issatchenkia terricola*, *Colletotrichum viniferum*, *Hanseniaspora vineae*, *Saprochaete gigas*, and *Candida diversa* represented the vast majority ofmicrobial species associated with sour rot-affected grapes. An in vitro spoilage assay confirmed that four of the isolated bacteria strains (two *Cronobacter* species, *Serratia marcescens* and *Lysinibacillus fusiformis*) and five of the isolated fungi strains (three *Aspergillus* species, *Alternaria tenuissima*, and *Fusarium proliferatum*) spoiled grapes. These microorganisms, which appear responsible for spoiling grapes in eastern China, appear closely related to microbes that cause this plant disease around the world.

## Introduction

Grape sour rot is a polymicrobial disease characterized by the disaggregation of the internal tissues of berries, detachment of the rotten berry from the pedicel, and a strong ethyl acetate smell. This disease causes millions of dollars in revenue loss each year due to decreases in the quality of berries (Barata et al., 2011; Steel, Blackman & Schmidtke, 2013). A number of microorganisms, such as *Ascomycota*, acetic acid bacteria (AAB), and filamentous fungi, can infect ripe and thin-skinned grape berries (Nally et al., 2013). Acetic acid released by AAB attracts the fruit fly Drosophila, which contributes to sour rot (Hall et al., 2018). The composition of microorganisms in sour rot-affected grapes depends on the cultivation site and grape variety.

The frequency and density of yeast species associated with sour rot differ between grape cultivars. The most frequently recovered ascomycetous species from rotten wine grapes are *Candida krusei*, *Kloeckera apiculata*, and *Metschnikowia pulcherrima* and a less frequent species is *Issatchenkia occidentalis* (Guerzoni & Marchetti, 1987). Barata et al. (2008) reported that *Candida vanderwaltii*, *Hanseniaspora uvarum*, and *Zygoascus hellenicus* are the most frequent species in rotten *Trincadeira Preta* red grape. The relative abundance of these microorganisms depends on the ripening stage and the availability of nutrients. Basidiomycetes and the yeast-like fungus *Aureobasidium pullulans* dominate intact grape berries. Ascomycetes with higher fermentative activity, like *Pichia* spp., *Zygoascus hellenicus*, wine spoilage yeasts, and AAB, are more frequent in rotten grape samples than in healthy grapes (Barata, Malfeito-Ferreira & Loureiro, 2012a; Barata, Malfeito-Ferreira & Loureiro, 2012b). Other than the widespread *Hanseniaspora uvarum* in sour rot wine grapes and table grapes, non-saccharomyces yeast (NSY) and AAB species occur in sour rot table grape. Pinto et al. (2017) proved that among all NSY-AAB associations, the yeastbacterium association composed of *Candida zemplinina* CBS 9494 and *Acetobacter syzygii* LMG 21419 shows the highest prevalence. This microbial consortium produces spoilage metabolites such as acetic acid and gluconic acid (Pinto et al., 2019).

Advances in molecular biology techniques and metagenomics have facilitated microbial community analyses (Andreote, Azevedo & Araújo, 2009) and characterization of microbes associated with plant diseases (Huang et al., 2017; Shen et al., 2018; Brady et al., 2017). Hall, O’Bryon & Osier (2019) characterized the microbiome of sour rot-affected grapes in New York by high-throughput sequencing and found that *Acetobacter* species were significantly more abundant in symptomatic samples than in asymptomatic ones. Studies of the microbial diversity of bacteria and fungi in rotten grapes in the eastern coast of China, a very important grape growing region, are limited.

In this study, metagenomic analysis was used to determine the diversity and abundance of bacteria and fungi in sour rot-affected table grapes collected from Yantai city. In parallel, we isolated several microbes and determined their potential to spoil grapes in an in vitro assay.

## Materials & Methods

### Samples of sour rot-affected grapes

Sour rot-affected grapes infested with fruit flies were collected from vineyards in Yantai (N36°27′, E117°10′), Shandong Province, China. Approximately 1.0 g of rotten tissue was sliced from each sour rot-affected grape (Muscat), and the tissues from 100 sour rot-affected grapes were collected together into one 50-mL sterile centrifuge tube. Three replicates from a total of 300 sour rot-affected grapes were stored at −80 °C for 16S rDNA and ITS high-throughput sequencing. Another three replicates were used for the separation and identification of culturable microorganisms in sour rot-affected grapes.

### 16S rDNA and ITS high-throughput sequencing analysis

#### DNA extraction and Illumina MiSeq sequencing of 16S rDNA and ITS genes

DNA was extracted from three rotten group samples using the Insect DNA Kit (OMEGA) and further purified using the MoBio PowerSoil Kit. The bacterial universal primers 341 F (5′-CCTACACGACGCTCTTCCGATCTN (barcode) CCTACGG-GNGGCWGCAG-3′) and 805 R (5′-GACTGGAGTTCCTTGGCACCCGAGAA- -TTCCA (barcode) GACTACHVGGGTATCTAATCC-3′) were used for amplification of the V3–V4 region of 16S rDNA. The fungal universal primers ITS4 F (5′-CCCTACACGACGCTCTTCCGATCTN (barcode) TCCTCCGCTTATTGATATG-3′) and ITS3 R (5′-GTGACTGGAGTTCCTTGG CACCCGAGAATTCCAGCATCGAT- -GAAGAACG–CAGC-3′) were used for amplification of the ITS gene. Each reaction comprised 15 µL of Phurs Mix (2er), 1.5 µL of each primer, 10 ng of template DNA, and ddH_2_O. The cycling conditions were as follows: initial denaturation at 95 °C for 1 min, followed by 35 cycles at 95 °C for 10 s, 54 °C for 30 s, and 72 °C for 30 s, and a final extension at 72 °C for 5 min. ITS was amplified under similar cycling conditions, except for the annealing temperature (52 °C). The sequencing libraries for 16S rDNA and ITS were constructed using the TruSeq DNA PCR-Free Preparation Kit (Illumina, San Diego, CA, USA) and quantified using Qubit 3.0 (Life Technologies, Grand Island, NY, USA). Then, the library was sequenced on an Illumina MiSeq platform (HiSeq 2000; PE250). After the removal of low-quality reads and primer/adaptor sequences using SeqClean, high-quality reads (clean data) were generated and used for further analysis. These sequencing procedures were performed by Sangon Biotech (Shanghai, China) Co., Ltd.

#### Alpha diversity analysis

Sequences were clustered into operational taxonomic units (OTUs) using the 97% identity threshold (3% dissimilarity level) (Mothur, https://mothur.org/) (Schloss et al., 2009). The reads of 16S rDNA and ITS sequences had been submitted in the Short Read Archive (BioProject ID: PRJNA61015). According to the number of OTUs, the rarefaction curve for 16S rDNA and ITS sequences was made to measure the adequacy and rationality of data. Shannon and Simpson diversity indexes were calculated as indicators of microbial diversity, and Chao1 and ACE indices were calculated as indicators of microbial richness using Mothur (Schloss et al., 2009). All OTUs were analyzed using BLASTN and the 16S rDNA and ITS databases (http://ncbi.nlm.nih.gov/). The best results (similarity >90% and coverage>90%) were used for subsequent classification. The sequences that did not satisfy these criteria were defined as “unclassified.” Species richness and relative abundances were estimated.

### Diversity of culturable microorganisms in sour rot-affected grapes

The above samples with three replicates were suspended in phosphate-buffered saline (PBS, 0.2 M, pH 7.2) at ratios of 1:10^3^, 1:10^4^ and 1:10^5^. The suspension (200 µL; different concentrations) was spread on nutrient agar medium and potato dextrose agar medium with three replicates each. After culturing at 25 °C for 48 h on nutrient agar and 7 d on potato dextrose agar a total of 80 colonies were picked and restreaked for purity using the primary media.

#### Identification of cultivated bacteria

Physiological and biochemical characteristics of each bacterium were analyzed according to the methods described by  Dong & Cai (2001). The following tests were performed: Gram staining, spore staining, bacterial motility test, catalase reaction, methyl red test, starch hydrolysis, benzopyrrole test, VP test, malonic acid test, gelatin test, H_2_S test, citrate test, ammonia production test, litmus milk test, and urease test.

DNA was extracted from a single colony of each bacterium using the Bacterial DNA Kit (OMEGA, Norcross, GA, USA) and purified using the DNA Clean-Up Kit (OMEGA). 16S rDNA was amplified for each DNA template using the Bio-Rad 1000-Series Thermal Cycler PCR (Hercules, CA, USA). The thermal cycling profile was as follows: initial denaturation at 95 °C for 3 min, followed by 35 cycles of 95 °C for 15 s, 54 °C for 30 s, and 72 °C for 1 min, and a final extension at 72 °C for 5 min. Primer sequences were as follows: 16S rDNA-27F: 5′-AGAGT TTGATCCTGGCTCAG-3′; 16S rDNA-1492R: 5′-TACGGYTACCTTGTTACGACTT-3′.

#### Identification of cultivated fungi

The morphological features of each fungus were analyzed using a light microscope (CX41RF; Olympus, Tokyo, Japan) according to the methods described by Dai (1978). The mycelium of each purified fungus was collected in PDA medium. DNA was extracted using the Fungal DNA Kit (OMEGA) and purified using the DNA Clean-Up Kit (OMEGA). The ITS gene was amplified according to the following thermal cycling profile: initial denaturation at 95 °C for 3 min, quantification for 35 cycles (95 °C for 15 s followed by 52 °C for 30 s and 72 °C for 1 min), and a final extension at 72 °C for 5 min. Sequences of the universal primers were as follows: ITS1: 5′-TCCGTAGGTGAACCTGCGG-3′; ITS4: 5′-TCCTCCGCTTATTGATATGC-3′.

#### Sequencing

PCR products were purified using the TaKaRa Mini BEST Agarose Gel DNA Extraction Kit (Takara, Japan) and sequenced on an ABI-3730 DNA analyzer (Applied Biosystems, Foster City, CA, USA).The sequences were analyzed using BLAST (http://ncbi.nlm.nih.gov/). Phylogenetic trees of bacteria and fungi were separately constructed using the neighbor-joining method (NJ; Saitou & Nei, 1987) implemented in MEGA 6.0 (LynnonBiosoft, San Ramon, CA, USA). The sequences of bacteria and fungi were submitted to GenBank using SEQUIN (see phylogenetic trees for accession numbers).

### Spoilage potential assay of cultivated bacteria and fungi

Isolated bacteria and fungi were tested for spoilage potential on grape berries. Healthy grape berries of Midnight Beauty, a susceptible variety, were collected and surface sterilized with 1% sodium hypochlorite (NaClO) solution for one minute. Excess NaClO was removed by washing the berries twice in sterile distilled water. The experimental berries were pricked 2–3 mm deep using a dissecting needle to simulate the wounds made by fruit flies during egg laying or other mechanical damage. The bacterial suspension and fungal spore suspension were prepared with a concentration of approximately 1 × 10^6^ cfu/ml or conidia/ml in the suspension. This suspension (5 µl per berry) was used to inoculate the wounds of healthy grape berries. Sterile water was used instead of the suspension as a negative control. Two methods were used to observe the spoilage potential of bacteria and fungi. In the merged placement method, 10 grape berries were placed in a single Petri dish (10 cm in diameter and three cm in height) to simulate grape clusters in the field. In the separate placement method, each of 10 grape berries was placed in a single culture bottle (2.5 cm in diameter and three cm in height). Three replicates were established for both methods and each bacterium and fungus treatment. Subsequently, the inoculated grape berries were kept in a moisture chamber at 27 °C/25 °C (day/night) and 80% humidity, and symptoms were recorded on the 5th day. The bacterial and fungal species were reisolated from these artificially inoculated grape berries using NA medium and PDA medium, respectively. The resulting culture was compared with the original culture (Hyun et al., 2001).

Based on the ratio of the infected area to the total area, grading was performed as follows (Rouxel et al., 2013; Zhou et al., 2014): 0, no disease spot; 1, less than 5% of the total area infected; 3, 5% to 25% of the total area infected; 5, 25% to 50% of the total area infected; 7, 50% to 75% of the total area infected; 9, 75% to 100% of the total area infected. The incidence (%) and the McKinney index of bacteria and fungi were calculated according to the following formulas:

(1) \begin{eqnarray*}\mathrm{The} \text{percentage} \mathrm{of} \text{incidence} (\text{%})& =100\ast \frac{\mathrm{the} \text{number} \mathrm{of} \text{diseased} \text{berries}}{\mathrm{the} \text{number} \mathrm{of} \mathrm{all} \text{berries}} \end{eqnarray*}

(2) \begin{eqnarray*}\mathrm{The} \text{McKinney} \text{index}& =100\ast \sum _{k=0}^{\mathrm{n}} \frac{kx}{9N} \end{eqnarray*}

where, *x* is the value for each grade; *n* is the number of diseased berries at each level; and *N* is the total number of fruits investigated.

## Results

### Sequencing and alpha diversity analyses

The mean lengths of 16S rDNA of bacteria and ITS gene of fungi generated from metagenomic DNA extracted from rotting grapes were 413 ± 3 bp and 279 ± 5 bp, respectively. The OTU numbers for bacteria and fungi were 1,343 ± 283 and 1,039 ± 387 respectively (Table 1). Rarefaction curves of three samples for 16S rDNA and ITS sequences reached asymptotes (Fig. S1), which suggests coverage was sufficient. The number of sequences for each OTU decreased rapidly by the OTU rank of 18 (Fig. S1). The flat curve indicated a high degree of sequencing uniformity.

**Table 1:** Sequence information of bacterium and fungi in sour rot-affected grapes.

Group	Sample	Number of raw reads	Mean length of raw reads	Number of clean reads	Mean length of clean reads	Number of filtered reads
16S rDNA	1	56,220	447	54,080	410	38,313
	2	63,599	458	61,916	418	39,939
	3	52,692	449	51,690	410	24,500
	Mean ± SE	57,504 ± 3,213	451 ± 3	55,895 ± 3,088	413 ± 3	
ITS	1	80,740	317.03	80,628	2,746	79,658
	2	71,362	332	71,281	2,886	71,160
	3	76,531	318	76,432	276	76,154
	Mean ± SE	76,211 ± 2,712	322 ± 5	76,114 ± 2,704	2,794 ± 5	

In a phylogenetic tree of the top 50 bacterial OTUs, 15 OTUs were classified as phylum *Firmicutes*, class *Bacilli*. Among the other 35 *Proteobacteria*, 21 OTUs belonged to the class *Alphaproteobacteria*, three to *Betaproteobacteria*, and 11 to *Gammaproteobacteria* (Fig. S2). In a phylogenetic tree of the top 50 fungal OTUs, one belonged to Basidiomycota and eight were not identified based on searches against the ITS database. Among the other 41 *Ascomycota* OTUs, 29 belonged to the class *Saccharomycetes*, nine to *Sordariomycetes*, and two to *Dothideomycetes* (Fig. S3).

The microbial diversity, as determined by the Shannon index and Simpson index, was higher for bacteria than fungi, and richness, as determined by the Chao1 index and ACE index, was higher for fungi than bacteria (Table 2).

**Table 2:** Diversity indices of bacterium and fungi in sour rot-affected grapes.

Parameters	Parameters	Bacterium (Mean ± SE)	Fungi (Mean ± SE)
Diversity indices	Shannon	3 ± 1.3 E–01	220 E–2 ± 1.7 E–01
	ACE	22,034 ± 2,927	32,667 ± 1385
	Chao1	9,745 ± 1,430	10,779 ± 1476
	Simpson	1 E–01 ± 2 E–02	21 E-2 ± 4 E–02
OTUs number		1,343 ± 283	1,039 ± 386

### Microbial taxonomic analysis

**Figure 1:**
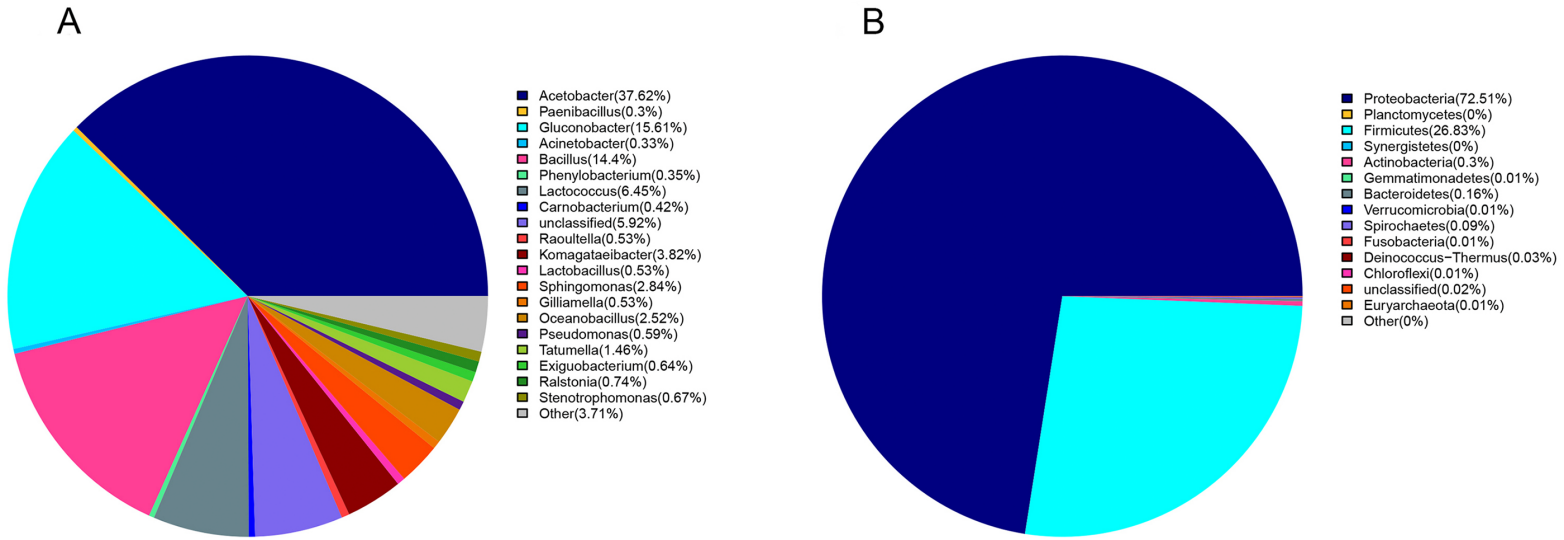
The bacterial community structure in sour rot-infected grapes based on 16S rDNA high-throughput sequencing. (A) The bacterial community structure based on genus; (B) the bacterial community structure based on phylum.

*Proteobacteria* (72%) and *Firmicutes* (27%) were dominant among the 19 bacteria phyla identified (Fig. 1A). The proportion of other bacteria was less than 1%. The dominant genera in sour rot-affected grapes were *Acetobacter* (38%), *Gluconobacter* (24%), *Bacillus* (12%), and *Lactococcus* (Fig. 1B).

**Figure 2:**
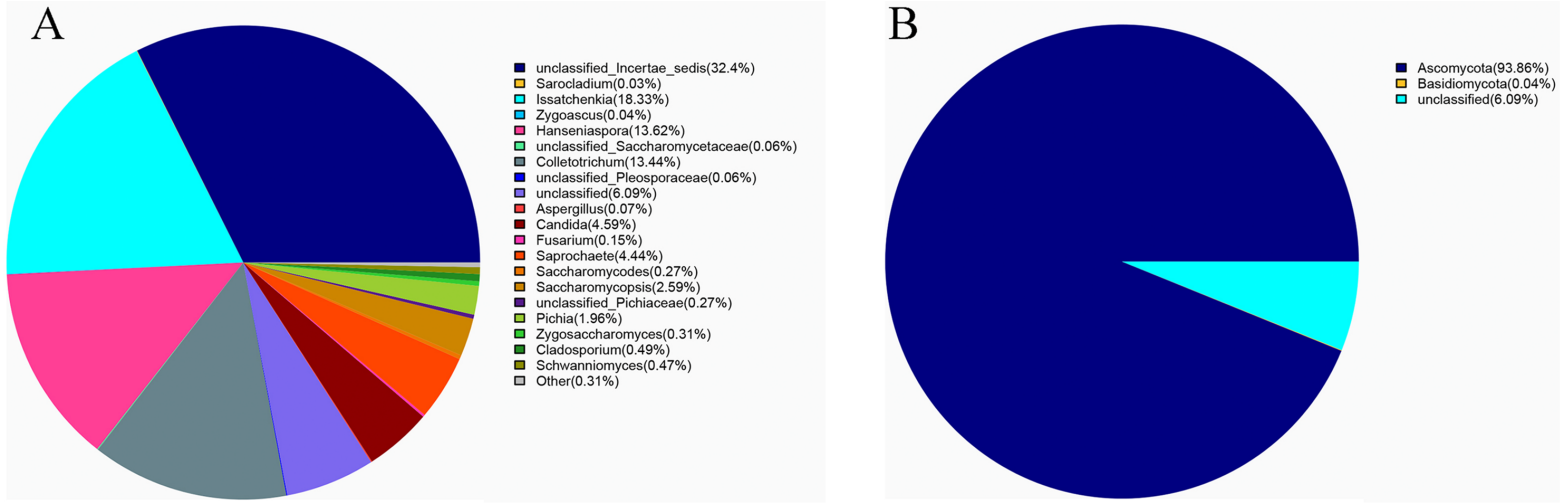
The fungal community structure in sour rot-affected grapes based on ITS high-throughput sequencing. (A) The fungal community structure based on genus; (B) the fungal community structure based on phylum.

*Ascomycota* (94%) was the dominant phylum in the identified fungal community (Fig. 2A). The dominant species identified in sour rot-affected grapes were *Issatchenkia terricola* (18%), *Colletotrichum viniferum* (13%), *Hanseniaspora vineae* (13%), *Saprochaete gigas* (4%), and *Candida diversa* (4%), and 32% of isolates were not taxonomically identified (*Incertae sedis* sp.) (Fig. 2B).

### Diversity of culturable microorganisms in sour rot-affected grapes

We cultured 15 bacterial strains from sour rot-affected grapes infested by fruit flies (Table 3). We identified *Firmicutes* as the dominant phylum (60%), with nine Gram-positive bacteria species (i.e., *Staphylococcus saprophyticus*, *Lactococcus garvieae*, *Lactobacillus plantarum*, two *Lysinibacillus* species, and four *Bacillus* species). We also isolated six Gram-negative bacteria species assigned to the phylum *Proteobacteria*. All bacterial taxa presented positive results in catalase reaction, gelatin, H_2_S, and ammonia production assays whereas they presented negative results for fermentation with the methyl red. Strains classified as *Cronobacter malonaticus*, *Cronobacter sakazakii*, and *Klebsiella pneumoniae* presented negative results for these biochemical tests (Table 4).

**Table 3:** Phylogeny of microbes isolated from sour rot-affected grapes.

Microorganism	Phylum	Species	Strain IDs	Accession numbers
Bacterium	*Proteobacteria*	*Cronobacter malonaticus*	SRG1	MK743990
		*Cronobacter sakazakii*	SRG2	MK743989
		*Klebsiella pneumoniae*	SRG3	MK743987
		*Acetobacter* sp.	SRG4	MK743980
		*Serratia marcescens*	SRG5	MK743984
		*Enterobacter hormaechei*	SRG6	MK743988
	*Firmicutes*	*Staphylococcus saprophyticus*	SRG7	MK743982
		*Lactococcus garvieae*	SRG8	MK743983
		*Lactobacillus plantarum*	SRG9	MK743986
		*Lysinibacillus fusiformis*	SRG10	MK753026
		*Lysinibacillus* sp.	SRG11	MK743985
		*Bacillus amyloliquefaciens*	SRG12	MK743994
		*Bacillus cereus*	SRG13	MK743993
		*Bacillus* sp.*-* 1	SRG14	MK743992
		*Bacillus* sp.*-* 2	SRG15	MK743991
Fungus	*Deuteromycotina*	*Cladosporium oxysporum*	SRG16	MK748311
		*Alternaria tenuissima*	SRG17	MK748314
		*Saprochaete gigas or Geotrichum gigas*	SRG18	MN567950
		*Fusarium proliferatum*	SRG19	MK748309
		*Nigrospora* sp.	SRG20	MK748317
	*Ascomycotina*	*Penicillium citrinum*	SRG21	MK748316
		*Penicillium georgiense*	SRG22	MK748315
		*Aspergillus niger*	SRG23	MK748313
		*Aspergillus oryzae*	SRG24	MK748312
		*Aspergillus aculeatus*	SRG25	MK748310

**Table 4:** The physiological and biochemical characteristic of bacterium in sour rot-affected grape.

Bacterium	Strain IDs	Gram staining	Spore staining	Bacterial motility	Catalase reaction	Methyl red test	Starch hydrolysis test	Benzpyrole test	V-P test
*Cronobacter malonaticus*	SRG1	–		–	+	–	–	–	+
*Cronobacter sakazakii*	SRG2	–		–	+	–	–	–	+
*Klebsiella pneumoniae*	SRG3	–		–	+	–	–	–	+
*Acetobacter* sp.	SRG4	–		+	+	–	–	–	+
*Serratia marcescens*	SRG5	–		+	+	–	+	–	+
*Enterobacter hormaechei*	SRG6	–		+	+	–	+	–	+
*Staphylococcus saprophyticus*	SRG7	+		+	+	–	+	–	+
*Lactococcus garvieae*	SRG8	+		–	+	–	+	–	+
*Lactobacillus plantarum*	SRG9	+		–	+	–	+	–	+
*Lysinibacillus fusiformis*	SRG10	+	purple	+	+	–	+	–	–
*Lysinibacillus* sp.	SRG11	+	purple	+	+	–	+	–	+
*Bacillus amyloliquefaciens*	SRG12	+	pink	+	+	–	+	–	–
*Bacillus cereus*	SRG13	+	purple	+	+	–	+	–	+
*Bacillus* sp.-1	SRG14		purple	+	+	–	+	–	+
*Bacillus* sp.-2	SRG15	+	purple	+	+	–	+	–	+

Bacterium		Malonic acid test	Gelatin test	H_2_S test	Citrate test	Ammonia production test	Litmus milk test	Urease test
*Cronobacter malonaticus*	SRG1	–	+	+	+	+	+	–
*Cronobacter sakazakii*	SRG2	+	+	+	–	+	+	–
*Klebsiella pneumoniae*	SRG3	+	+	+	+	+	–	+
*Acetobacter* sp.	SRG4	+	+	+	+	+	+	+
*Serratia marcescens*	SRG5	+	+	+	–	+	+	–
*Enterobacter hormaechei*	SRG6	+	+	+	+	+	+	–
*Staphylococcus saprophyticus*	SRG7	–	+	+	+	+	+	–
*Lactococcus garvieae*	SRG8	–	+	+	–	+	+	–
*Lactobacillus plantarum*	SRG9	+	+	+	+	+	+	–
*Lysinibacillus fusiformis*	SRG10	+	+	+	–	+	–	–
*Lysinibacillus* sp.	SRG11	+	+	+	+	+	–	–
*Bacillus amyloliquefaciens*	SRG12	–	+	+	+	+	+	+
*Bacillus cereus*	SRG13	–	+	+	+	+	+	–
*Bacillus* sp.-1	SRG14	+	+	+	+	+	+	+
*Bacillus* sp.-2	SRG15	–	+	+	+	+	+	+

**Figure 3:**
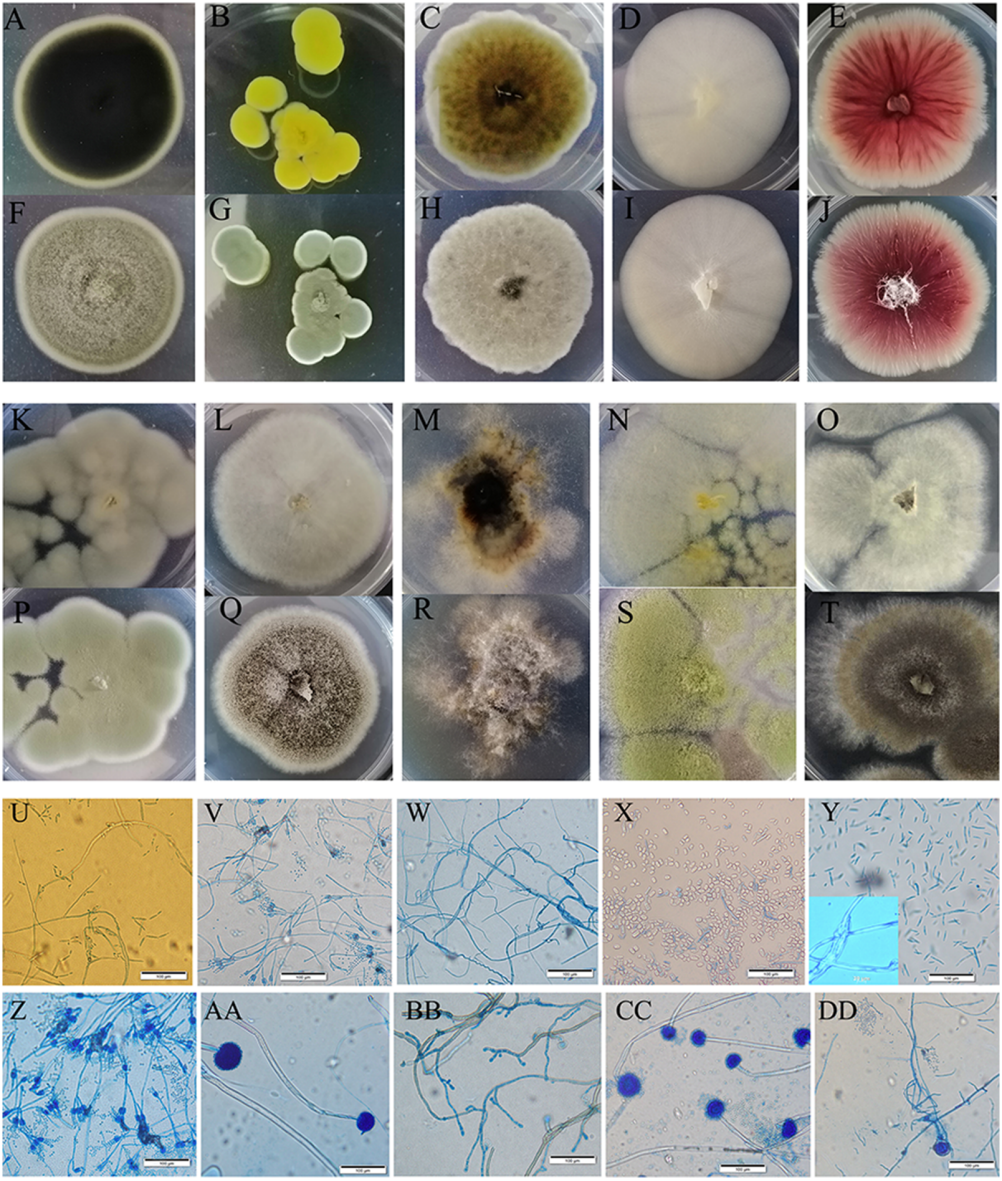
Colony morphology and the light mophology of the fungi in sour rot-affected grapes. (A–E) represent the reverse side of colony morphology of *Cladosporium oxysporum* , *Penicillium citrinum*, *Alternaria tenuissima*, *Saprochaete gigas*, *Fusarium proliferatum*; (F–J) represent the front side of colony morphology of *Cladosporium oxysporum*, *Penicillium citrinum*, *Alternaria tenuissima*, *Saprochaete gigas*, *Fusarium proliferatum*; (K–O) represent the reverse side of colony morphology of *P. georgiense*, *Aspergillus niger*, *Nigrospora* sp., *A. oryzae*, *A. aculeatus*; (P–T) represent the font side of colony morphology of *P. georgiense*, *Aspergillus niger*, *Nigrospora* sp., *A. oryzae*, *A. aculeatus*. (U–DD) represent the light morphology of *Cladosporium oxysporum*, *Alternaria tenuissima*, *Saprochaete gigas*, *Fusarium proliferatum*, *Nigrospora* sp*.*, *Penicillium citrinum*, *P. georgiense*, *Aspergillus niger*, *A. oryzae*, *A. aculeatus*.

We cultured ten fungi from sour rot-affected grapes. Five were classified as Deuteromycotina, including *Cladosporium oxysporum*, *Alternaria tenuissima*, *Geotrichum gigas*, *Fusarium proliferatum*, and *Nigrospora* sp. (Table 3). *C. oxysporum*, with bottle-green colonies, developed into conidia by asexual reproduction. *A. tenuissima* colonies, with a white front side and brown reverse side, developed into conidia in the form of a chain lattice. The hyphae of *Saprochaete gigas* or *Geotrichum gigas*, with white colonies, developed into arthrospores by asexual reproduction. *F. proliferatum*, with red colonies, had branched conidiophores and sickle or long column-shaped conidia. *Nigrospora* sp. had irregular colonies, branched conidiophores, and ball-shaped conidia. Five species (i.e., *Penicillium citrinum*, *P. georgiense*, *Aspergillus niger*, *A. aculeatus*, and *A. oryzae*) belonged to Ascomycotina. The sporophores of *P. citrinum* and *P. georgiense* grew from hyphae and developed into brush-like structures. These two *Penicillium* species differed in colony color. The conidia of *A. niger*, *A. aculeatus*, and *A. oryzae* were black, green, and yellow, respectively (Fig. 3).

### Spoilage potential of culturable bacteria and fungi for grape sour rot

**Figure 4:**
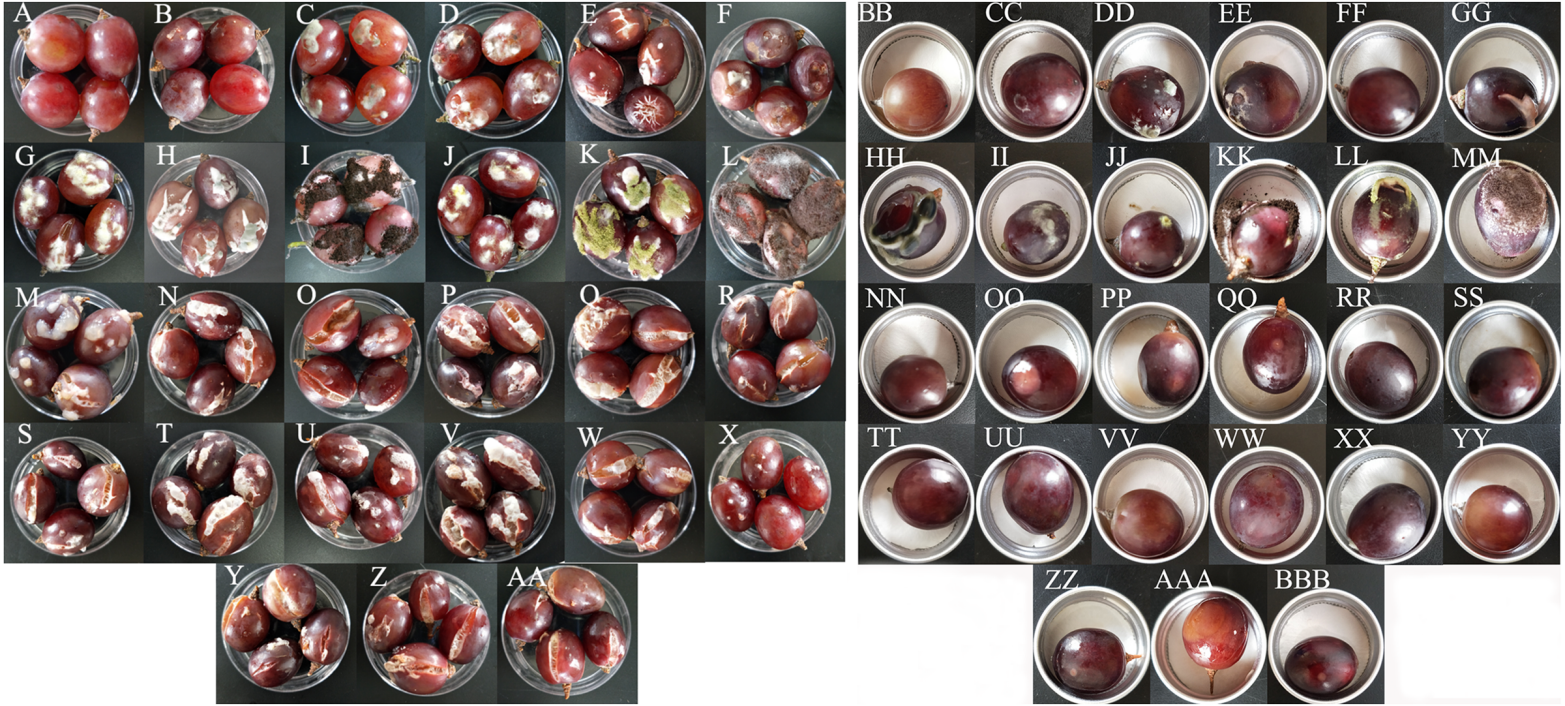
The pathogenicity of bacteria and fungi in healthy grape berries. (A–AA) represent pathogenicity of sterile water, LB medium, *Cladosporium oxysporum*, *Alternaria tenuissima*, *Saprochaete gigas*, *Fusarium proliferatum*, *Nigrospora* sp., *Penicillium citrinum*, *P. georgiense*, *Aspergillus niger*, *A. oryzae*, *A. aculeatus*, *Cronobacter malonaticus*, *C. sakazakii*, *Klebsiella pneumoniae*, *Acetobacter* sp., *Serratia marcescens*, *Enterobacter hormaechei*, *Staphylococcus saprophyticus*, *Lactococcus garvieae*, *Lactobacillus plantarum*, *Lysinibacillus fusiformis*, *Lysinibacillus* sp., *Bacillus amyloliquefaciens*, *B. cereus*, *Bacillus* sp.*-* 1, *Bacillus* sp.*-* 2 using the merged method; (BB–BBB) represent pathogenicity of sterile water, LB medium, *Cladosporium oxysporum*, *Alternaria tenuissima*, *Saprochaete gigas*, *Fusarium proliferatum*, *Nigrospora* sp., *Penicillium citrinum*, *P. georgiense*, *Aspergillus niger*, *A. oryzae*, *A. aculeatus*, *Cronobacter malonaticus*, *C. sakazakii*, *Klebsiella pneumoniae*, *Acetobacter* sp., *Serratia marcescens*, *Enterobacter hormaechei*, *Staphylococcus saprophyticus*, *Lactococcus garvieae*, *Lactobacillus plantarum*, *Lysinibacillus fusiformis*, *Lysinibacillus* sp., *Bacillus amyloliquefaciens*, *B. cereus*, *Bacillus* sp.*-* 1, *Bacillus* sp.*-* 2 using the separated method.

All 15 bacterial species and 10 fungal species demonstrated the potential to spoil grapes. In the merged placement method, all of the microorganisms except for *B. amyloliquefaciens*, caused cracking and infection in grapes (Fig. 4A). In the separate placement method, all of fungi except for *Saprochaete gigas*, could cause infection in grapes. Obvious symptoms were not detected in the grapes treated by bacterium (Fig. 4B). The bacterial species and the fungal species reisolated from these spoiled grapes using NA medium and PDA medium were confirmed as the original microorganisms summarized in Table 3.

**Figure 5:**
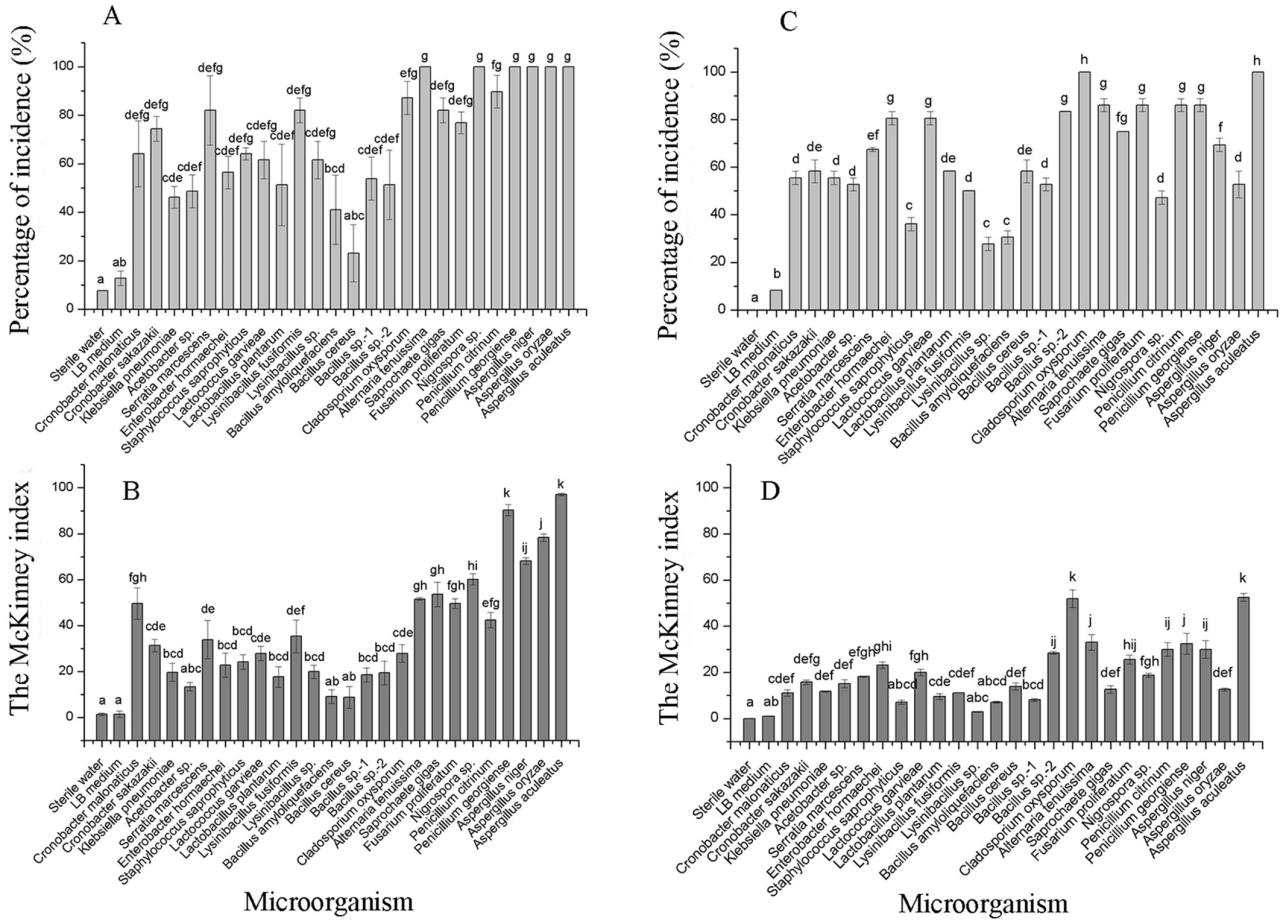
The percentage of incidence of microorganism using merged (A) or separated (C) methods; McKinney index of microorganism using merged (B) or separated (D) methods. Different letters in each figure indicate significant difference between microorganisms (one-way ANOVA; *α* = 0.05).

The incidence and McKinney index of 15 bacterial species and 10 fungal species were significantly different from those of the control (sterile water and LB medium) using the merged placement method (incidence: *F* = 10.44, *P* < 0.01; McKinney index: *F* = 43.28, *P* < 0.01; Fig. 5A and Fig. 5B). Fungal isolates demonstrated stronger spoilage potential in the grape berries with an incidence of more than 75%. Except for *C. oxysporum* and *P. citrinum*, the McKinney index of all other fungi exceeded 50%, which was greater than that of bacteria. Three *Aspergillus* species and *P. georgiense* showed 100% spoilage on the McKinney index. Healthy grapes were also highly sensitive to *A. tenuissima* and *F. proliferatum*, which high McKinney index values of 52 ± 1 and 50 ± 2, respectively. Among the bacteria, the incidence and McKinney index of two *Cronobacter* species, *Serratia marcescens* and *Lysinibacillus fusiformis*, were higher than those of the other bacteria. *B. amyloliquefaciens* and *B. cereus* led to less serious spoilage than other bacteria (Fig. 4, then Fig. 5). The percentages of incidence and McKinney index of microorganism were lower using separated infection method than merged method, while those of fungi were also higher than bacteria (Percentage of incidence: *F* = 90.52, *P* < 0.01; McKinney index: *F* = 50.49, *P* < 0.01; Fig. 5C and Fig. 5D). The *C. oxysporum* had the highest spoilage potential among microbial taxa, which was different from the results obtained under merged infection.

## Discussion

Metagenomic analysis and culturing indicated that the microorganisms caused spoilage were similar around the world. AAB were the dominant bacteria in rot-affected grapes in eastern coastal areas of China, which is consistent with reports from Australia (Mateo et al., 2014), Portugal (Barata, Malfeito-Ferreira & Loureiro, 2012b) and New York (Hall, O’Bryon & Osier, 2019). *Aspergillis* were the dominant mold, which agrees with reports from Greece (Tjamos et al., 2004) and California (Rooney-Latham et al., 2008). *Issatchenkia occidentalis*, *Hanseniaspora uvarum*, and *Candida vanderwaltii*, *Colletotrichum viniferum*, and *Saprochaete gigas*, were also commonly observed, which are the same genera, but different species, than reports from other regions (Guerzoni & Marchetti, 1987; Barata et al., 2008; Barata, Malfeito-Ferreira & Loureiro, 2012b; Lleixa et al., 2018). Pisani, Nguyen & Gubler (2015) reported that grape sour rot is a disease complex involving many filamentous fungi and bacteria but is usually initiated by *A. niger* or *A. carbonarius* in California. We observed similar communities and reported the presence of pathogens, that could infect humans and animals, associated with rotting grapes, such as *C. sakazakii* SRG2, *K. pneumoniae* SRG3, and *S. gigas* SRG18. *C. sakazakii* is an emerging opportunistic foodborne pathogen with the potential to cause meningitis, bacteremia, and necrotizing enterocolitis, particularly in infants (Drudy et al., 2006; Aly et al., 2019). *K. pneumoniae* is an important conditional pathogenic and iatrogenic infectious bacterium. *Saprochaete* yeasts have emerged as fungal pathogens and causal agents of life-threatening infections in patients with severe neutropenia and hematological malignancies (Pavone et al., 2019). Therefore, sour rotten berries could be a reservoir for human pathogens.

Fungal isolates demonstrated greater spoilage potential than bacterial isolates in the grape berries. Except for three *Aspergillus* species with high McKinney index values, healthy grapes were also sensitive to spoilage fungi (*A. tenuissima* and *F. proliferatum*) associated with common grape diseases, which was different from studies in other places. As the most common species in the cosmopolitan genus *Alternaria*, *A. tenuissima* is found on a broad range of fruit products and causes various diseases, like post-harvest black rot of fruit (Logrieco, Moretti & Solfrizzo, 2009). Bakshi, Sztejnberg & Yarden (2001) reported that *F. proliferatum* could also cause the rot of corn, rice, and lily. Therefore, *Aspergillus* species, *A. tenuissima,* and *F. proliferatum* were the main cultivated spoilage fungi causing sour rot in grapes*.* Among the bacterial isolates, *B. amyloliquefaciens* and *B. cereus* led to less serious sour rot in this study. This can possibly be explained by the antibacterial substances generated by *B. amyloliquefaciens* and *B. cereus*, which have been used as biological control agents (Risoen, Ronning & Hegna, 2004; Wang et al., 2014). Although *L. fusiformis* restricts the biofilm formation of some pathogenic bacteria, it caused serious rot in grape berries (Fig. 3). Healthy grapes were sensitive to *Cronobacter* sp. and *S. marcescens*, which are spoilage microorganisms (Healy et al., 2010). The spoilage potential assay confirmed that *Cronobacter* species, *S. marcescens*, and *L. fusiformis* can cause sour rot in grapes. In this study, the incidence and McKinney index of microorganisms were lower using the separate infection method than using the merged method, further suggesting that diseases related to sour rotten grapes could spread quickly through grape clusters.

Sour rot is the culmination of coinfection by various yeasts that convert grape sugars to ethanol and bacteria that oxidize the ethanol to acetic acid (Pinto et al., 2019), and *Drosophila* spp. mediate these processes (Hall et al., 2018). Sour rot increases attractiveness to ovipositing *D. melanogaster* females and oviposition by *D. suzukii* facilitates sour rot development (Rombaut et al., 2017; Ioriatti et al., 2018). Furthermore, musts and the beginning of fermentation using rotten Macabeo grapes is consistently characterized by an elevated frequency of Zygosaccharomyces, and AAB increase in the late stages of fermentation (Lleixa et al., 2018). It is difficult to control sour rot in grapes due to the multiple species associated with this disease. Therefore, relationships among insects, microorganisms, and grapes as well as comprehensive analyses of nosogenesis will be the key question in the researches of sour rot in grapes.

## Conclusions

This study identified more spoilage species in sour rot-affected grapes of China using culture-dependent methods combined with high-throughput sequencing analysis, which would provide comprehensive information on targets for the control of the disease. Majority of these microbes could infect grapes with wounds. The microbes associated with sour grape rot in eastern coastal China appear similar to those associated with this disease in vineyards around the world. We reported here that *A. tenuissima*, and *F. proliferatum* spoil grapes. Human and animal pathogens were also present among the bacteria in sour rot-affected grapes, such as *Cronobacter sakazakii*, *Klebsiella pneumoniae* and *S. gigas*.

##  Supplemental Information

10.7717/peerj.9376/supp-1 Table S1 Raw data for spoilage potential of microorganism using two methods The McKinney index and percentage of incidence for spoilage potential of microorganism with three replicates using merged and separated methods

10.7717/peerj.9376/supp-2 Figure S1 The rarefaction curves and abundance-OUT rank curves of 16S rRNA and ITS sequences based on 16S rDNA high-throughput sequencing The rarefaction curves****and abundance-OUT rank curves of 16S rRNA and ITS sequences based on 16S rDNA high-throughput sequencing. The curves of three samples tended to be flat in rarefaction curves, which indicated that the amount of sequential data of three samples were reasonable. The flat curves of abundance-OUT rank indicated a high degree of sequencing uniformity

10.7717/peerj.9376/supp-3 Figure S2 The first 50 OTUs of the bacteria by high-throughput sequencing The first 50 OTUs of the bacteria by high-throughput sequencing

10.7717/peerj.9376/supp-4 Figure S3 The first 50 OTUs of the fungi by high-throughput sequencing

10.7717/peerj.9376/supp-5 Data S1 The raw data of 16S rDNA through high throughput sequencing from 5’ end The original image data file of 16S rDNA obtained by Illumina Miseq™ from 5’ end was transformed into the original sequencing sequence by CASAVA Base Calling analysis (Sequenced Reads).

10.7717/peerj.9376/supp-6 Data S2 The raw data of 16S rDNA through high throughput sequencing from 3’ end The original image data file of 16S rDNA obtained by Illumina Miseq™ from 3’ end was transformed into the original sequencing sequence by CASAVA Base Calling analysis (Sequenced Reads).

10.7717/peerj.9376/supp-7 Data S3 The raw data of ITS through high throughput sequencing The original image data file of ITS gene obtained by Illumina Miseq™ was transformed into the original sequencing sequence by CASAVA Base Calling analysis (Sequenced Reads).

10.7717/peerj.9376/supp-8 Data S4 Sequences of bacteria in sour rotten grape

10.7717/peerj.9376/supp-9 Data S5 Sequences of fungi in sour rotten grape

## Additional Information and Declarations

### DNA Deposition

The following information was supplied regarding the deposition of DNA sequences: The group sequences of bacteria are available at GenBank: MK743980 to MK743994 and MK753026. The group sequences of fungi are available at GenBank: MK748309 to MK748317. The Saprochete gigas sequence is available at GenBank: MN567950.

### Data Availability

The following information was supplied regarding data availability: The raw measurements are available in the Supplementary Files.
