# Supplementary material for: Diversity and spoilage potential of microbial communities associated with grape sour rot in eastern coastal areas of China
Source: PeerJ. 2020 Jun 16;8:e9376. doi: 10.7717/peerj.9376 (PMC7315622; doi:10.7717/peerj.9376)
Supplement: Table S1 — The McKinney index and percentage of incidence for spoilage potential of microorganism with three replicates using merged and separated methods [file peerj-08-9376-s001.docx]

| **Raw data for spoilage potential of microorganism using two method** | | | | | | | |
| --- | --- | --- | --- | --- | --- | --- | --- |
| Methods | Microorganism | The McKinney index | | | Percentage of incidence (%) | | |
|  |  | Replication 1 | Replication 2 | Replication 3 | Replication 1 | Replication 2 | Replication 3 |
| Merged method | Sterile water | 2.56 | 0.85 | 0.85 | 7.69 | 7.69 | 7.69 |
|  | LB medium | 0 | 0 | 4.27 | 0.00 | 0.00 | 38.46 |
|  | *Cronobacter malonaticus* | 63.08 | 44.62 | 41.03 | 84.62 | 69.23 | 38.46 |
|  | *Cronobacter sakazakii* | 29.06 | 28.21 | 36.75 | 69.23 | 69.23 | 84.62 |
|  | *Klebsiella pneumoniae* | 27.35 | 14.53 | 17.09 | 53.85 | 38.46 | 46.15 |
|  | *Acetobacter sp.* | 17.09 | 11.97 | 11.11 | 61.54 | 46.15 | 38.46 |
|  | *Serratia marcescens* | 30.77 | 21.37 | 49.57 | 92.31 | 53.85 | 100.00 |
|  | *Enterobacter hormaechei* | 33.33 | 17.95 | 17.09 | 69.23 | 53.85 | 46.15 |
|  | *Staphylococcus saprophyticus* | 23.93 | 18.8 | 29.91 | 61.54 | 61.54 | 69.23 |
|  | *Lactococcus garvieae* | 22.22 | 28.21 | 33.33 | 46.15 | 69.23 | 69.23 |
|  | *Lactobacillus plantarum* | 26.5 | 13.68 | 12.82 | 84.62 | 30.77 | 38.46 |
|  | *Lysinibacillus fusiformis* | 27.35 | 49.57 | 29.06 | 76.92 | 92.31 | 76.92 |
|  | *Lysinibacillus sp.* | 25.68 | 17.95 | 16.24 | 76.92 | 53.85 | 53.85 |
|  | *Bacillus amyloliquefaciens* | 14.53 | 8.55 | 4.27 | 69.23 | 30.77 | 23.08 |
|  | *Bacillus cereus* | 10.26 | 16.24 | 0 | 30.77 | 38.46 | 0.00 |
|  | *Bacillus sp.-1* | 23.08 | 12.82 | 19.66 | 53.85 | 38.46 | 69.23 |
|  | *Bacillus sp.-2* | 26.5 | 9.4 | 22.22 | 69.23 | 23.08 | 61.54 |
|  | *Cladosporium oxysporum* | 20.51 | 33.33 | 29.91 | 76.92 | 100.00 | 84.62 |
|  | *Alternaria tenuissima* | 52.14 | 52.14 | 50.43 | 100.00 | 100.00 | 100.00 |
|  | *Fusarium proliferatum* | 53.85 | 47.86 | 47.01 | 84.62 | 76.92 | 69.23 |
|  | *Nigrospora sp.* | 60.68 | 64.1 | 55.56 | 100.00 | 100.00 | 100.00 |
|  | *Penicillium citrinum* | 44.44 | 35.9 | 47.01 | 92.31 | 76.92 | 100.00 |
|  | *Penicillium georgiense* | 94.87 | 86.32 | 89.74 | 100.00 | 100.00 | 100.00 |
|  | *Aspergillus niger* | 67.52 | 65.81 | 70.94 | 100.00 | 100.00 | 100.00 |
|  | *Aspergillus oryzae* | 81.2 | 77.78 | 76.07 | 100.00 | 100.00 | 100.00 |
|  | *Saprochaete gigas or Geotrichum gigas* | 53 | 44.44 | 63.25 | 76.92 | 76.92 | 92.31 |
|  | *Aspergillus aculeatus* | 96.58 | 96.58 | 98.29 | 100 | 100 | 100 |
| Separated method | Sterile water | 0 | 0.00 | 0.00 | 0 | 0.00 | 0.00 |
|  | LB medium | 0.93 | 0.93 | 0.93 | 8.33 | 8.33 | 8.33 |
|  | *Cronobacter malonaticus* | 10.19 | 13.89 | 9.26 | 58.33 | 58.33 | 50.00 |
|  | *Cronobacter sakazakii* | 16.67 | 13.89 | 16.67 | 66.67 | 58.33 | 50.00 |
|  | *Klebsiella pneumoniae* | 12.04 | 11.11 | 12.04 | 58.33 | 50.00 | 58.33 |
|  | *Acetobacter sp.* | 18.52 | 12.96 | 13.89 | 50.00 | 50.00 | 58.33 |
|  | *Serratia marcescens* | 18.52 | 18.52 | 17.59 | 66.67 | 66.67 | 69.23 |
|  | *Enterobacter hormaechei* | 25.93 | 22.22 | 21.30 | 83.33 | 83.33 | 75.00 |
|  | *Staphylococcus saprophyticus* | 5.56 | 7.41 | 8.33 | 33.33 | 33.33 | 41.67 |
|  | *Lactococcus garvieae* | 20.37 | 22.22 | 17.59 | 83.33 | 83.33 | 75.00 |
|  | *Lactobacillus plantarum* | 8.33 | 12.04 | 8.33 | 58.33 | 58.33 | 58.33 |
|  | *Lysinibacillus fusiformis* | 11.11 | 11.11 | 11.11 | 50.00 | 50.00 | 50.00 |
|  | *Lysinibacillus sp.* | 3.41 | 2.78 | 2.78 | 33.33 | 25.00 | 25.00 |
|  | *Bacillus amyloliquefaciens* | 6.48 | 7.41 | 7.41 | 25.00 | 33.33 | 33.33 |
|  | *Bacillus cereus* | 13.89 | 16.67 | 11.11 | 58.33 | 66.67 | 50.00 |
|  | *Bacillus sp.-1* | 9.26 | 7.41 | 7.59 | 50.00 | 50.00 | 58.33 |
|  | *Bacillus sp.-2* | 29.63 | 27.78 | 27.78 | 83.33 | 83.33 | 83.33 |
|  | *Cladosporium oxysporum* | 53.70 | 57.41 | 44.44 | 100.00 | 100.00 | 100.00 |
|  | *Alternaria tenuissima* | 29.63 | 39.81 | 29.63 | 83.33 | 91.67 | 83.33 |
|  | *Fusarium proliferatum* | 12.04 | 10.19 | 15.74 | 75.00 | 75.00 | 75.00 |
|  | *Nigrospora sp.* | 22.22 | 25.93 | 28.70 | 83.33 | 83.33 | 91.67 |
|  | *Penicillium citrinum* | 20.37 | 18.52 | 17.59 | 50.00 | 50.00 | 41.67 |
|  | *Penicillium georgiense* | 24.07 | 34.26 | 31.48 | 83.33 | 91.67 | 83.33 |
|  | *Aspergillus niger* | 39.81 | 33.33 | 24.07 | 91.67 | 83.33 | 83.33 |
|  | *Aspergillus oryzae* | 22.22 | 34.26 | 33.33 | 66.67 | 75.00 | 66.67 |
|  | *Saprochaete gigas or Geotrichum gigas* | 12.04 | 13.89 | 12.04 | 58.33 | 58.33 | 41.67 |
|  | *Aspergillus aculeatus* | 55.55 | 50.00 | 51.85 | 100.00 | 100.00 | 100.00 |
