# Supplementary figures and images for: Diversity and spoilage potential of microbial communities associated with grape sour rot in eastern coastal areas of China
Source: PeerJ. 2020 Jun 16;8:e9376. doi: 10.7717/peerj.9376 (PMC7315622; doi:10.7717/peerj.9376)

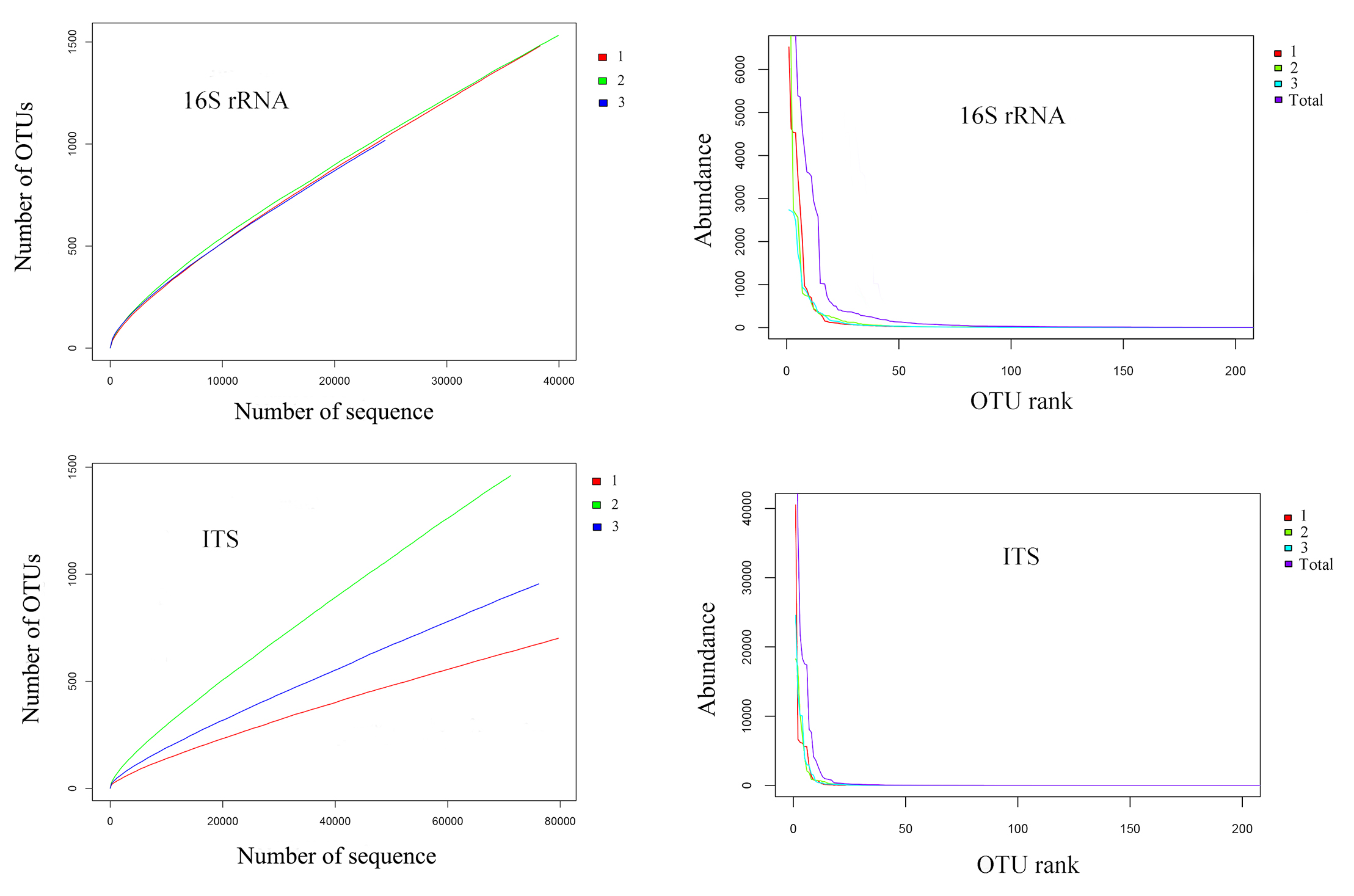

Supplement: Figure S1 — The rarefaction curvesand abundance-OUT rank curves of 16S rRNA and ITS sequences based on 16S rDNA high-throughput sequencing. The curves of three samples tended to be flat in rarefaction curves, which indicated that the amount of sequential data of three samples were reasonable. The flat curves of abundance-OUT rank indicated a high degree of sequencing uniformity [file peerj-08-9376-s002.jpg]

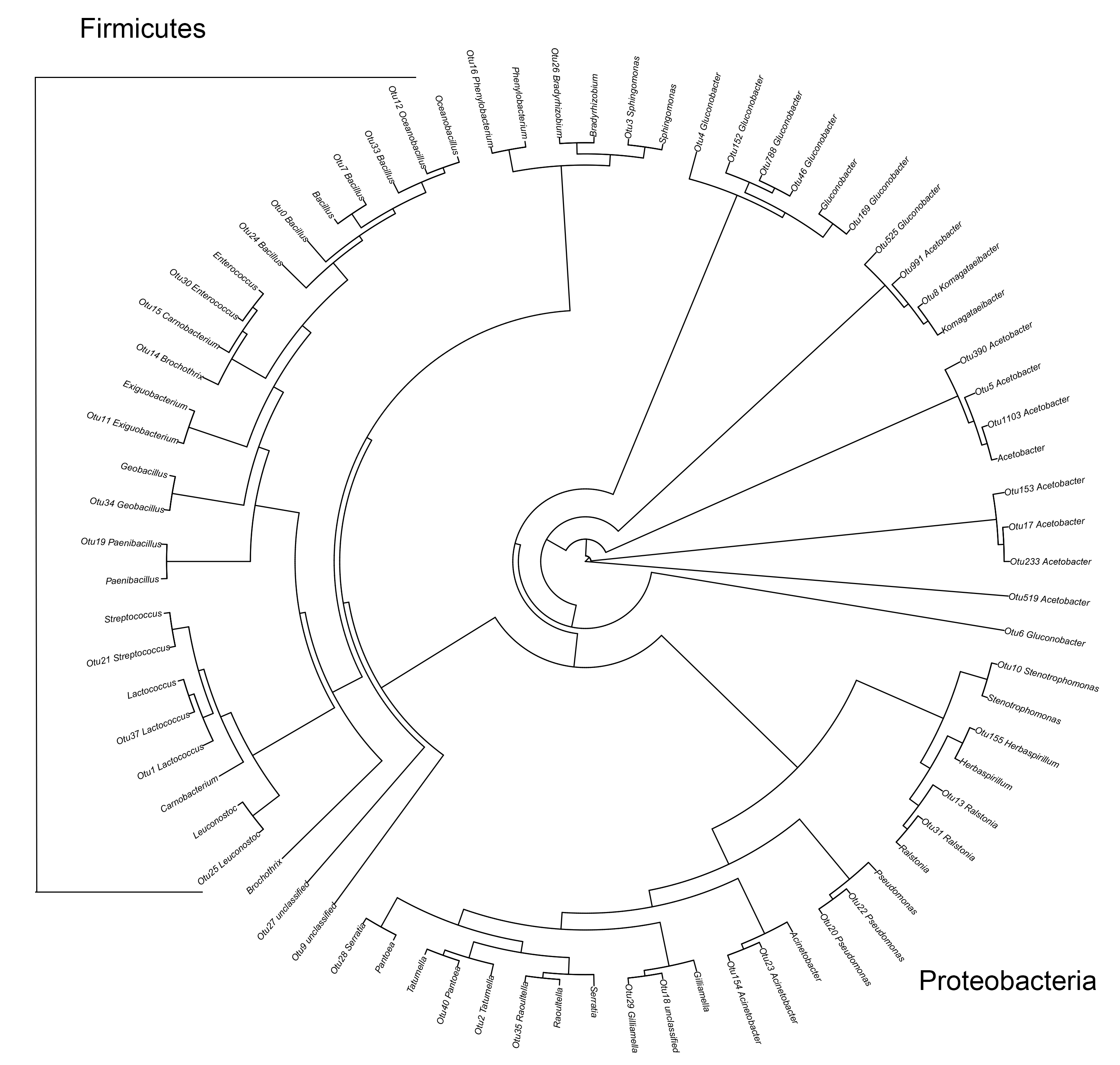

Supplement: Figure S2 — The first 50 OTUs of the bacteria by high-throughput sequencing [file peerj-08-9376-s003.jpg]

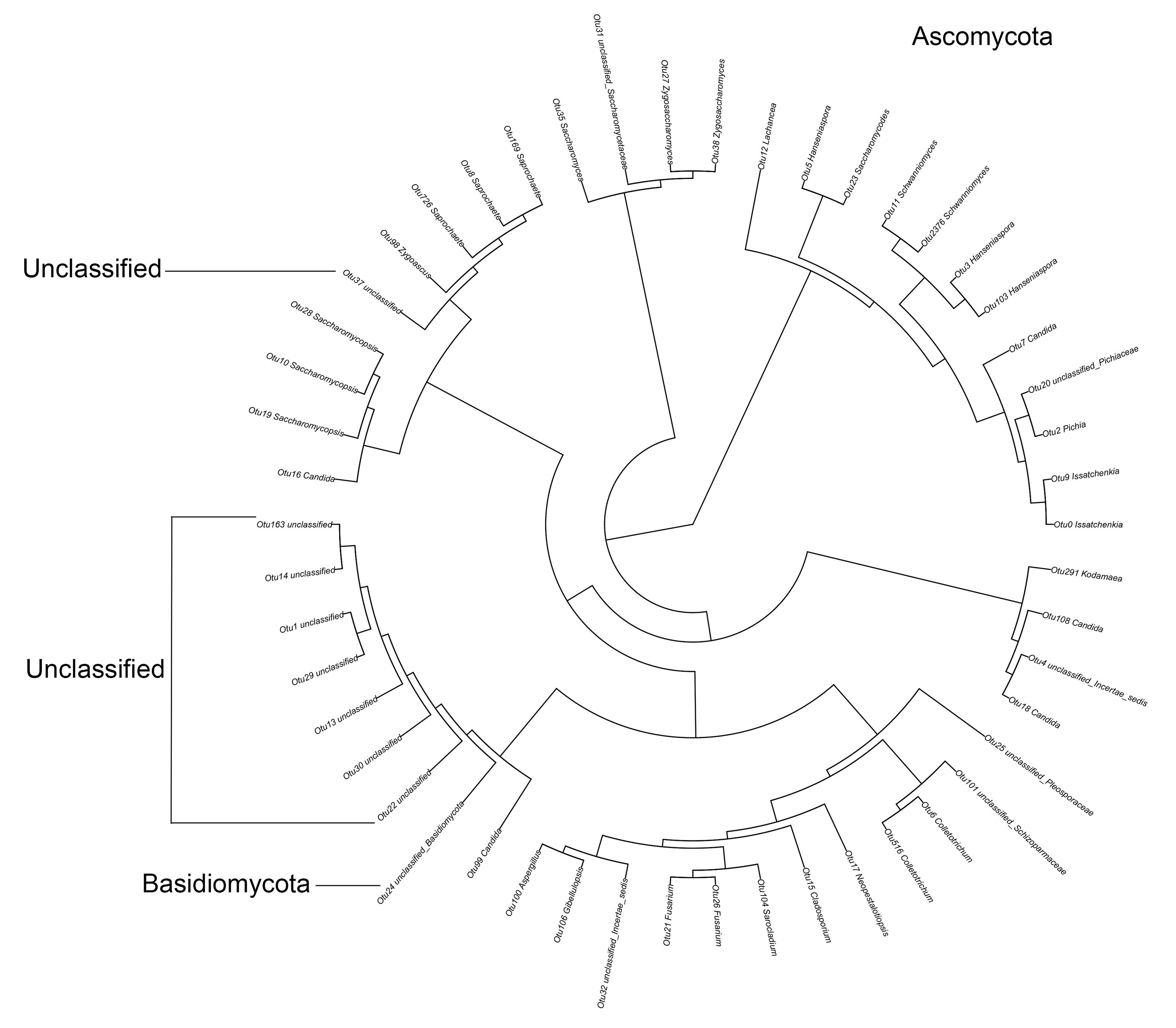

Supplement: Figure S3 [file peerj-08-9376-s004.jpg]
